# *Lactobacillus acidophilus* and its metabolite ursodeoxycholic acid ameliorate ulcerative colitis by promoting Treg differentiation and inhibiting M1 macrophage polarization

**DOI:** 10.3389/fmicb.2024.1302998

**Published:** 2024-01-16

**Authors:** Song Deng, Chaoying Pei, Kaiwei Cai, Wenyi Huang, Xiaoyi Xiao, Xingyuan Zhang, Rongyao Liang, Yanlong Chen, Zhiyong Xie, Pei Li, Qiongfeng Liao

**Affiliations:** ^1^School of Pharmaceutical Sciences, Guangzhou University of Chinese Medicine, Guangzhou, China; ^2^School of Pharmaceutical Sciences (Shenzhen), Sun Yat-sen University, Shenzhen, China

**Keywords:** *Lactobacillus acidophilus*, ulcerative colitis, ursodeoxycholic acid, Tregs, M1 macrophages, RapGap/PI3K-AKT/NF-κB pathway

## Abstract

*Lactobacillus acidophilus* (LA) is a common clinical probiotic that improves ulcerative colitis (UC) by restoring intestinal immune balance. However, the interaction of LA with the gut microbiota and its metabolites in the treatment of UC remains unknown. Therefore, this study seeks to elucidate whether the gut microbiota and its metabolites act as pivotal effectors in LA’s therapeutic mechanisms and how precisely they modulate intestinal immunity. In this study, we verified that LA can obviously ameliorate the disease severity, and regulate intestinal immune disorders in UC mice. Subsequently, antibiotic (ABX)-mediated depletion of the gut microflora demonstrated that the therapeutic efficiency of LA was closely associated with gut microbiota. In addition, the results of metabolomics revealed that ursodeoxycholic acid (UDCA), a metabolite of intestinal flora, may be a potential effector molecule mediating therapeutic effects of LA. Indeed, we found that UDCA can improve the macro pathological characteristics of UC mice, and through a comprehensive set of *in vivo* and *in vitro* experiments, we discovered that UDCA exerts dual effects on immune regulation. Firstly, it promotes the differentiation of Treg cells, resulting in increased secretion of anti-inflammatory cytokines. Secondly, UDCA inhibits the polarization of M1 macrophages, effectively reducing the secretion of pro-inflammatory cytokines. Moreover, we found that UDCA regulation of immune response is directly related to the RapGap/PI3K-AKT/NF-κB signaling pathway. In conclusion, LA and its metabolite, UDCA, may treat UC by activating the RapGap/PI3K-AKT/NF-κB signaling pathway and modulating Treg cells and M1 macrophages. All in all, our findings highlight the potential of microbial metabolites in enhancing probiotic for UC treatment.

## Graphical abstract

LA elevates UDCA levels in UC mice, and together they mitigate intestinal inflammation in ulcerative colitis by modulating the RapGap/PI3K-AKT/NF-κB signaling pathway and ameliorating intestinal immune disorders.
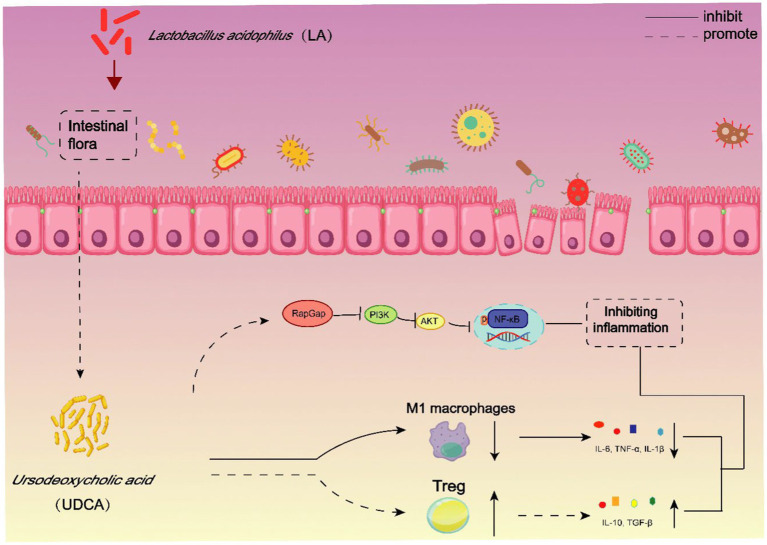

## 1 Introduction

Ulcerative colitis (UC) is characterized by mucosal erosions and intestinal tract bleeding, which leads to disability and severe body weight loss (Ungaro et al., 2017). The incidence of UC is closely related to socio-economic status, with a much higher prevalence in developed countries than in developing ones (Buie et al., 2023). Although the exact etiology of UC remains unclear, there is considerable evidence that the disease results from a combination of genetic, environmental, and dietary factors (Nakase et al., 2022). Concurrently, gut microbial imbalance and abnormal intestinal mucosal immune regulation are also associated with the occurrence and development of UC (Ma et al., 2021).

Intestinal flora plays a crucial role in the occurrence and development of UC through two ways: (1) direct effects involving interactions with the host’s microbiota through recognition receptors, resulting in downstream molecular and functional changes; and (2) indirect effects on the host through various metabolites, influencing host immunity, mucosal barrier, and energy metabolism. Regulating intestinal flora to modulate metabolite secretion, receptor recognition, and host immunity is essential for alleviating UC. Among the immune cells involved in UC pathogenesis, macrophages and T helper (Th) cells have received significant attention (Yao et al., 2019). Macrophages exist in distinct functional states: classically activated (M1) macrophages drive pro-inflammatory responses, while alternatively activated (M2) macrophages exert anti-inflammatory functions. In parallel, recent investigations have unveiled alterations in the T helper cell profile in clinical UC patients, characterized by an increase in T helper cell 17 (Th17) cells accompanied by a reduction in regulatory T cells (Tregs) (Long et al., 2020). The dysregulated balance between Treg and Th17 subsets has been associated with UC pathogenesis, prompting external interventions to restore their equilibrium as a potential therapeutic avenue. In summary, targeting macrophage polarization and Treg/Th17 balance with drugs offer promising therapeutic interventions for UC.

Furthermore, bile acids (BAs), essential microbial metabolites, have emerged as pivotal modulators of gut immune homeostasis. Recent studies have illuminated the effects of specific BAs, such as 3-oxo-lithocholic acid and isoallo-lithocholic acid, in driving differentiation of Th17 and Treg cells within the intestinal lamina propria (Hang et al., 2019). In addition, lithocholic acid (LCA) exerts its anti-inflammatory effects by activating the Takeda G protein-coupled receptor 5 (TGR5) receptors, inducing the polarization of M1 macrophages towards M2 macrophages (Biagioli et al., 2017). Therefore, the involvement of BAs in modulating immune responses highlights their potential as therapeutic targets in UC management.

The primary treatment for UC involves anti-inflammatory drugs or immunosuppressive agents such as aminosalicylic acid, corticosteroids, and azathioprine. However, these drugs have significant adverse effects, and long-term use may cause severe complications. At present, probiotics, which have received widespread attention, can not only effectively avoid the above-mentioned disadvantages, but also have excellent performance in regulating intestinal flora and host immunity (Shen et al., 2022; Xu et al., 2022; Zheng et al., 2023). At the same time, *Lactobacillus acidophilus* (LA), which our research group has been concerned about for a long time, has not only been proved to play an anti-UC effect by regulating intestinal flora, but also has a potential function of regulating intestinal immunity, although its mechanism *in vivo* is still unclear (Wang et al., 2020; Li P. et al., 2022). Sulfasalazine (SASP) is a commonly used drug for the treatment of inflammatory intestinal diseases in clinical practice. Its main components are 5-amino-salicylic acid and sulfapyridine. The former usually has antibacterial and anti-inflammatory effects and immunosuppressive effects, while the latter has the effect of regulating intestinal flora (Mikami et al., 2023). Therefore, in the experimental design of this subject, we use SASP as a positive drug to investigate the efficacy of LA.

Thus, the primary objective of this study is to comprehensively investigate the mechanism through which LA alleviates UC by modulating the BAs-immune axis. First, we employed the intestinal microbiota depletion method and metabolomics to demonstrate that LA alleviates UC by regulating intestinal flora and UDCA. Next, we explored the impact of LA on immune cells in UC mice, such as Treg/Th17 cells and macrophages, using flow cytometry. Additionally, we assessed the ability of UDCA-treated UC mice to maintain intestinal mucosal barrier integrity, reduce inflammatory responses, and regulate immune disorders. Furthermore, *in vitro* experiments were conducted to directly assess the effect of UDCA on M1 macrophages and Treg cells. Finally, we identified through intestinal tissue transcriptomics that LA regulates Treg cells and M1 macrophages by activating the RapGap/PI3K-AKT/NF-κB signaling pathway. In conclusion, this study aims to explore in detail whether LA alleviates UC by affecting the interaction between BA and host immunity, and to provide new theoretical support and ideas for the further application of LA in the treatment of UC.

## 2 Materials and methods

### 2.1 Reagents and chemicals

Dextran sulfate sodium (DSS, 36–50 kDa) was obtained from MP Biomedicals (Irvine, CA, United States). LA was obtained from Guangdong Microbial Culture Collection Center (Guangdong, China). Ursodeoxycholic acid, Dimethyl sulfoxide, Lipopolysaccharides, Metronidazole and Ampicillin sodium salt were obtained from Sigma-Aldrich (St. Louis, MO, United States). Neomycin sulfate and Vancomycin were obtained from APExBIO (Houston, TX, United States). Naïve CD4^+^ T cell isolation kit mouse, AutoMACS rinsing solution, LS columns were obtained from Miltenyi Biotec (Cologne, German). Mouse 1 × Lymphocyte Separation Medium was obtained from DAKEWE (Shenzhen, China). Collagenase II, Collagenase IV, Deoxyribonuelease I (Dnase I) a were obtained from Solarbio (Beijing, China). The whole cell lysis assay kit was obtained from KeyGEN BioTECH (Jiangsu, China). Ultrapure RNA kit and BCA protein assay kit were obtained from CWBIO (Beijing, China). ChamQ SYBR qPCR Master Mix and Hiscript III RT SuperMix for qPCR were obtained from Vazyme (Nanjing, China). Universal two-step test kit and DAB color kit were obtained from ZSGB-BIO (Beijing, China). The GAPDH, Occludin, Mucin-2 (Muc-2) antibody were obtained from Abcam (Cambridge, MA, United States). NF-κB, p-NF-κB, Akt, p-Akt, PI3K and RapGap antibody were obtained from Cell Signaling TECHNOLOGY (Boston, MA, United States). p-PI3K was obtained from Abmart (Shanghai, China). Goat anti-rabbit IgG secondary antibody was obtained from Affinity (San Antonio, TX, United States). BSA Alibumin Fraction V (BSA) was obtained from BioFROXX (Einhausen, German). Difco Skin milk was obtained from BD (Franklin, United States). BV421-conjugated IL-17A, BV421-conjugated CD86, APC-conjugated CD206, FITC-conjugated CD4, APC-conjugated CD25, FITC-conjugated F4/80, APC750-conjugated CD45 were obtained from BioLegend (San Diego, CA, United States). PE-conjugated Foxp3 and eBioscience FOXP3/Transcription were obtained from Thermo Fisher Scientific (Waltham, MA, United States). Recombinant murine IFN-γ, IL-2, Recombinant human TGF-β1, Ultra-LEAF Purified anti-mouse CD3, CD28, Purified anti-mouse IL-4, IFN-γ were obtained from PeproTech (Rocky Hill, NJ, United States).

### 2.2 Animals and ethical statement

C57BL/6J mice (male, 18–22 g) were purchased from Zhuhai Bestest Biotechnology Co., Ltd. with License No. SCXK (YUE) 2020-0051 (Guangzhou, China) and raised under specific pathogen-free (SPF) conditions. The temperature was kept at 20°C–25°C, the light and dark were alternated for 12 h, the relative humidity was set at 60%–70%, and the sterile water and standard food were strictly maintained. The study was supervised and approved by the Animal Ethics Committee of Guangzhou University of Chinese Medicine (Approval No. ZYD-2022-024). Upon arrival, the mice were acclimatized for 1 week before induction of colitis and the mice wellbeing was monitored at least daily throughout the experiments.

### 2.3 Antibiotic treatment

To eliminate as much bacteria in the gut of SPF mice as possible, 4 antibiotics-ampicillin (1 g/L), neomycin (1 g/L), vancomycin (0.5 g/L), and metronidazole (1 g/L)-were mixed and dissolved in sterile distilled water in the final concentrations described earlier to target gram-positive and gram-negative bacteria broadly, and mice drink freely for 10 days. The same amount of sterile distilled water was given to mice in the Control group on the same schedule. Collect mouse feces, extract feces DNA, and use agarose gel electrophoresis to confirm the effectiveness of the antibiotic treatment.

### 2.4 DSS-induced murine experimental colitis and treatment

Experimental colitis was induced by providing mice with drinking water containing 3% (wt/vol) DSS for 7 days. Mice were monitored daily to ensure that they consumed an approximately equal volume of DSS-containing water.

**Figure 1:**
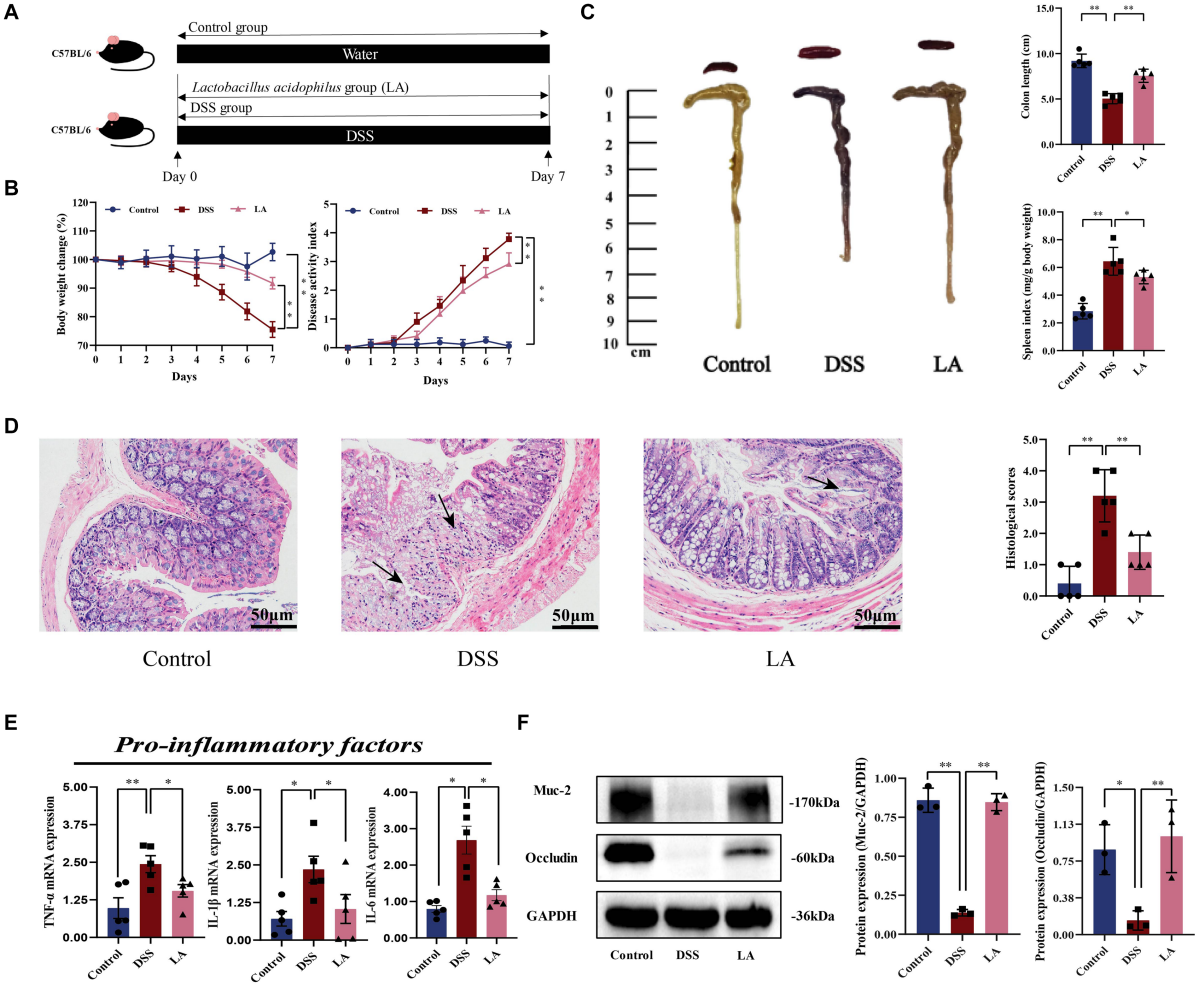
LA exerted protective effects and enhanced the repair of mucosal barrier function in DSS-induced ulcerative colitis. **(A)** Flow chart of animal experiment. **(B)** Body weight (shown as the percentage of initial body weight) and DAI were monitored every day (*n* = 5 mice per group). **(C)** Typical pictures of colon. Colon length (cm) and spleen index [expressed as spleen weight (mg)/body weight (g)] were showed (*n* = 5 mice per group). **(D)** Typical histological sections stained with H&E (200× magnification) and statistics of histological score (*n* = 5 mice per group) (inflammatory cell infiltration was indicated by black arrows). **(E)** The pro-inflammatory factors of TNF-α, IL-1β and IL-6 mRNA expression (*n* = 5 mice per group). **(F)** Western-blot assay of protein level of Muc-2, occludin. All data were presented as mean ± SD (*n* = 3 mice per group). ^*^p < 0.05 and ^**^p < 0.01. Control, mice treatment with sterile water; DSS, mice treatment with DSS; LA, mice treatment with DSS and *Lactobacillus acidophilus*.

For the first animal experiment, mice were randomly divided into 3 groups: Control (free to drink sterilized water and gavage PBS), DSS (free to drink DSS and gavage PBS), LA (free to drink DSS and gavage LA). The DSS group and LA group mice DSS modeling method as described previously. Mice in the Control group and DSS group were administrated 500 μL sterile water by gavage, while the LA group mice were administrated 500 μL LA (2 × 10^9^ colony-forming units (CFU)/mL) (day 1 to day 7) daily by gavage (Figure 1A).

**Figure 2:**
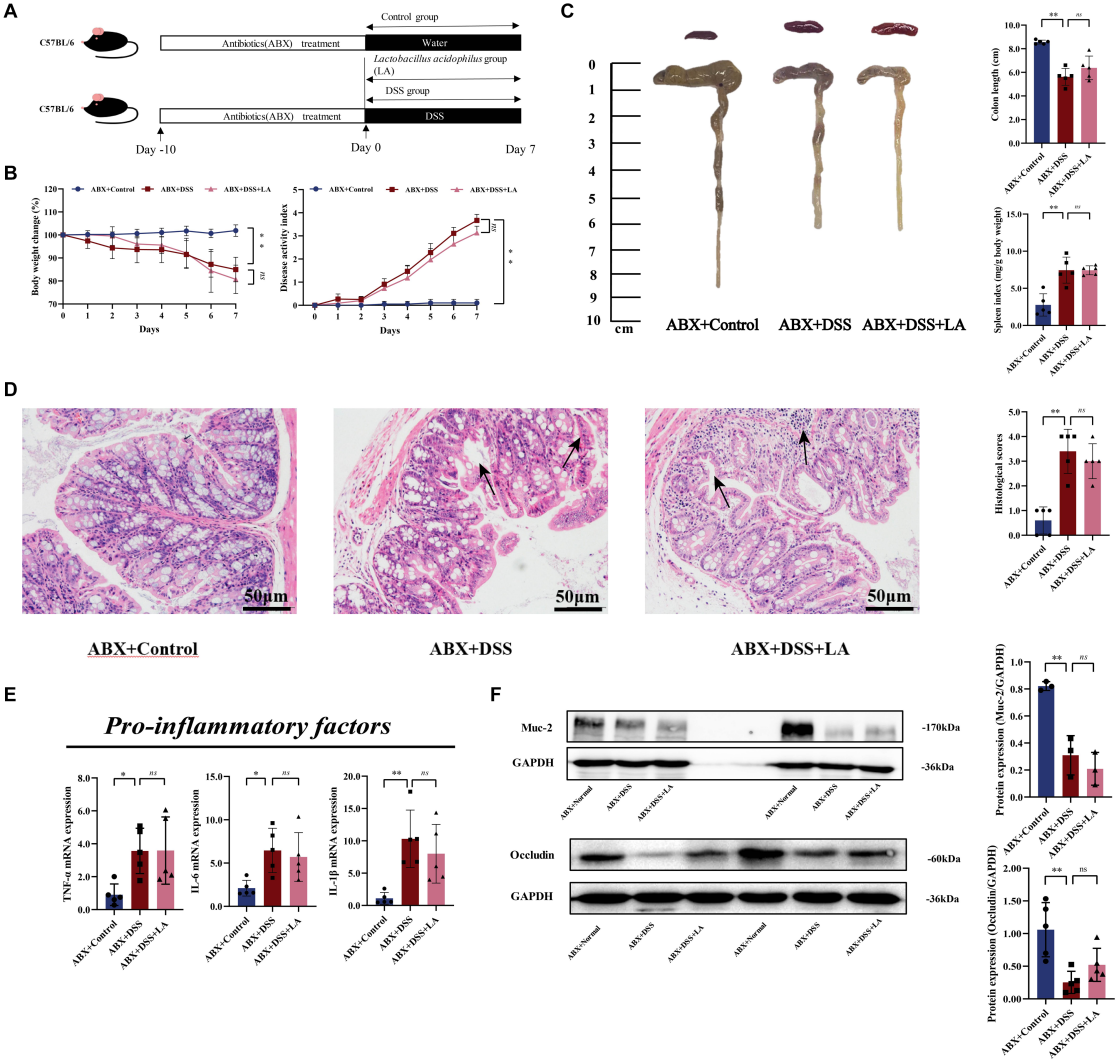
LA exerts anti-UC effects through intestinal flora. **(A)** Flow chart of animal experiment. **(B)** Body weight (shown as the percentage of initial body weight) and DAI were monitored every day (*n* = 5 mice per group). **(C)** Typical pictures of colon. Colon length (cm) and spleen index [expressed as spleen weight (mg)/body weight (g)] were showed (*n* = 5 mice per group). **(D)** Typical histological sections stained with H&E (200× magnification) and statistics of histological score (*n* = 5 mice per group) (inflammatory cell infiltration was indicated by black arrows). **(E)** The pro-inflammatory factors of TNF-α, IL-1β and IL-6 mRNA expression (*n* = 5 mice per group). **(F)** Western-blot assay of protein level of Muc-2, occludin (*n* = 3 mice per group). All data were presented as mean ± SD. ^*^p < 0.05 and ^**^p < 0.01. ^ns^*p* > 0.05.

For the second animal experiment, after confirming that gut bacteria were eliminated by antibiotics, mice were randomly divided into 3 groups after: ABX + Control (free drinking of sterilized water and gavage of PBS after clearing intestinal flora), ABX + DSS (free drinking of DSS and gavage of PBS after clearing intestinal flora), ABX + DSS + LA (free drinking of DSS and gavage of LA after clearing intestinal flora). DSS modeling methods as described previously, too. Mice in the ABX + Control group and ABX + DSS group were administrated 500 μL sterile water by gavage, while the ABX + DSS + LA group mice were administrated 500 μL LA (2 × 10^9^ CFU/mL) (day 1 to day 7) daily by gavage (Figure 2A).

**Figure 3:**
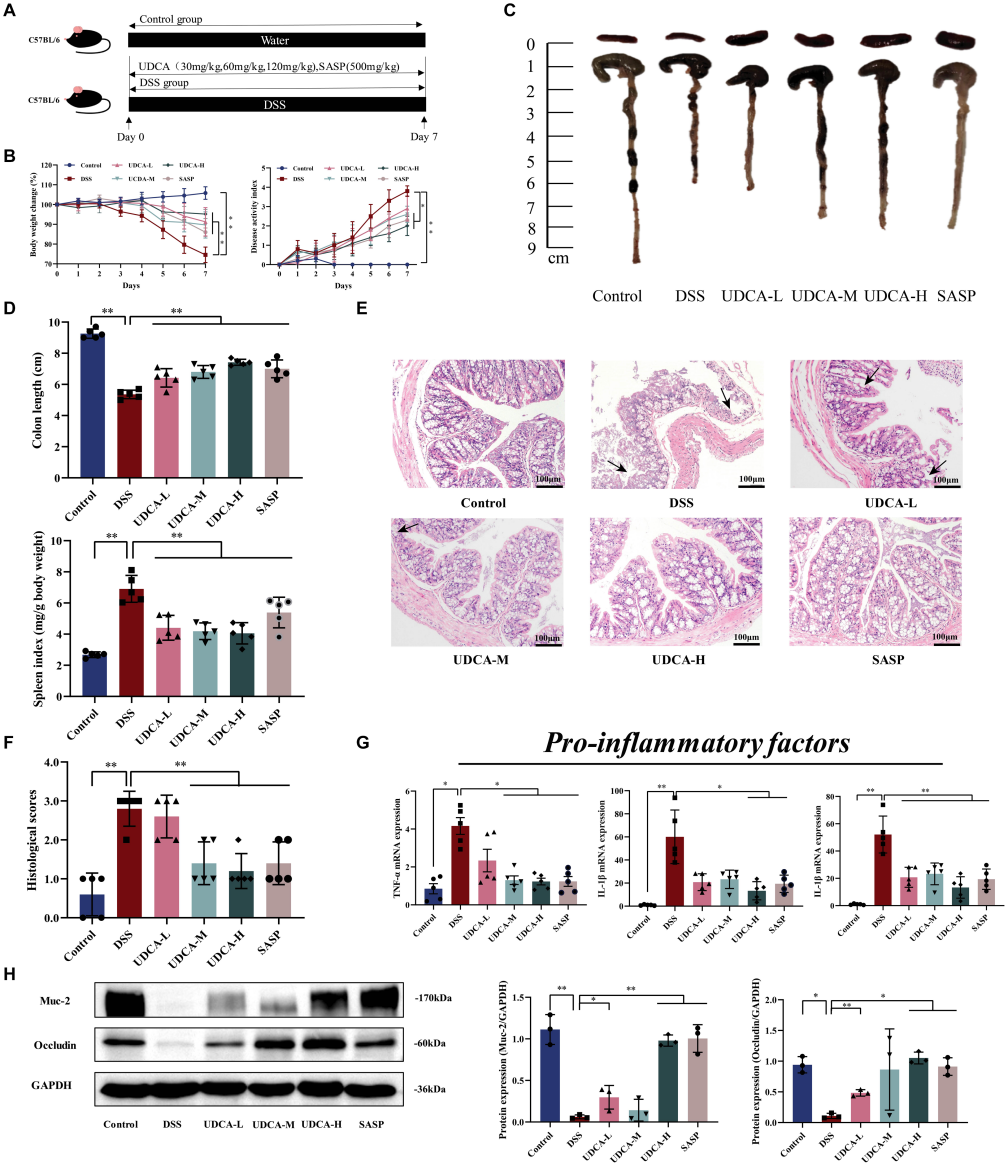
UDCA exerted protective effects and enhanced the repair of mucosal barrier function in DSS-induced ulcerative colitis. **(A)** Flow chart of animal experiment. **(B)** Body weight (shown as the percentage of initial body weight) and DAI were monitored every day (*n* = 5 mice per group). **(C)** Typical pictures of colon. **(D)** Colon length (cm) and spleen index [expressed as spleen weight (mg)/body weight (g)] were showed (*n* = 5 mice per group). **(E)** Typical histological sections stained with H&E (100× magnification). **(F)** Statistics of histological score (*n* = 5 mice per group) (inflammatory cell infiltration was indicated by black arrows). **(G)** The pro-inflammatory factors of TNF-α, IL-1β and IL-6 mRNA expression (*n* = 5 mice per group). **(H)** Western-blot assay of protein level of Muc-2, occludin. All data were presented as mean ± SD (*n* = 3 mice per group). ^*^p < 0.05 and ^**^p < 0.01. Control, mice treatment with sterile water; DSS, mice treatment with DSS; UDCA-L, M, H, mice treatment with DSS and UDCA.

For the third animal experiment, mice were randomly divided into 6 groups: Control, DSS, low-dose UDCA (UDCA-L), medium-dose UDCA (UDCA-M), high-dose UDCA (UDCA-H), Sulfasalazine (SASP). The DSS modeling methods as described previously. Mice in the Control group and DSS group were administrated 0.2 μL/10 g sterile water by gavage, mice in the UDCA-L, UDCA-M and UDCA-H groups were administration with UDCA (dissolved in sterile water, the dosage: 30 mg/kg, 60 mg/kg or 120 mg/kg) by gavage, mice in the SASP groups were administration with SASP (600 mg/kg) (day 1 to day 7) daily by gavage (Figure 3A).

### 2.5 Sample collection

After fasting for 16 h, the body weight of all animals was recorded, and the mice were treated with CO_2_ inhalation anesthesia. Subsequently, the blood samples were collected and centrifuged (3,000 g, 4°C) for 10 min, then, the upper serum was extracted and stored at −80°C. The colons were weighted, length measured and partly fixed in the 4% paraformaldehyde solution. The spleens were weighted. The contents of colon tract of mice were collected on ice and stored at −80°C.

### 2.6 Bacterial and cells culture

The probiotic strain of *Lactobacillus acidophilus* was cultured in De Man, Rogosa and Sharpe (MRS) agar or broth at 37°C, 5% CO_2_ for 48 h, the cultures were then centrifuged at 5,000 g for 10 min at room temperature. Bacteria were collected and the CFU of live LA were calculated by plate counting.

RAW264.7 cells were obtained from Guangzhou Jennio Biotech Co., Ltd. The cell lines were maintained in DMEM containing 10% Fetal Bovine Serum (FBS) and 1% penicillin-streptomycin (100 U/mL) at 37°C in a 5% CO_2_ incubator. The 6-well culture plates for RAW264.7 cells culture, and polarization towards to M1 macrophages with 250 ng/mL LPS and 20 ng/mL anti-IFN-γ 6 h in all groups except for the Control group. Then, pour out the medium, for the UDCA groups, 20 μM, 120 μM, 240 μM UDCA were added into the medium respectively, for the Control and 0 μM group, same amount of phosphate buffer saline (PBS) was added. All groups of cells were cultured under conditions of 5% CO_2_ and 37°C for 24 h prior to subsequent experiments.

### 2.7 Induction and analysis of Treg cells and treated with UDCA

Primary naive CD4^+^T cells were isolated from spleen of mice at the age of 6 to 8 weeks with Naive CD4^+^T Cell Isolation Kit mouse according to the manufacturer’s instructions. Naive CD4^+^T cells were cultured in RPMI 1640 medium (Sigma) supplemented with 10% FBS, penicillin-streptomycin (500 U), and β-mercaptoethanol (55 μM). The 96-well culture plates for Naive CD4^+^T cells culture were precoated with 10 μg/mL anti-CD3 mAb at 4°C overnight. Anti-CD28 mAb was added into the medium at 2 μg/μL concentration of polarization towards Treg cells was induced with 2 ng/mL recombinant human TGF-β1, 200 U/mL recombinant murine IL-2, 5 μg/mL anti-IL-4 and 5 μg/mL anti-IFN-γ. For the UDCA groups, 5 μM, 10 μM, 20 μM, 40 μM, 60 μM UDCA were added into the medium respectively, for the Control group, same amount of PBS was added. All groups of Cells were cultured under conditions of 5% CO_2_ and 37°C for 72 h prior to subsequent experiments.

### 2.8 Western blot

An equal amount of protein from each colonic sample was electrophoresed on SDS polyacrylamide gels with prestained protein markers. Proteins were then transferred to polyvinylidene difluoride (PVDF) membranes (Millipore, Billerica, MA, United States) and blocked in 5% fat-free powdered milk or BSA at room temperature in Tris-Tween-buffered saline (TBS: 20 mM Tris/150 mM NaCl, pH 7.5, and 0.1% Tween 20) for 3 h, the membranes were incubated separately with the following primary antibodies at 4°C overnight with gentle rocking: Muc-2 (1:1000), Occludin (1:1000), NF-κB (1:1000), p-NF-κB (1:1000), AKT (1:1000), p-AKT (1:1000), PI3K (1:1000), p-PI3K (1:2000) and RapGap (1:1000). Membranes were then washed and incubated with a horseradish peroxidase-conjugated secondary antibody at a 1:15000 dilution for 1 h at room temperature. Finally, using a ECL chemiluminescence kit, the blots were created, and the integrated density of pixels in each membrane was calculated with Image J software. The signal intensities were normalized to GAPDH.

### 2.9 RNA extraction and real-time quantitative PCR

Total RNA was extracted from a segment of the colon using Ultrapure RNA kit (CWBIO, Jiangsu, China), and 1 mg RNA was reverse-transcribed using a Hiscript III RT SuperMix kit (Vazyme, Jiangsu, China). Real-time quantitative PCR (RT-qPCR) was conducted on the analytic-jena qTOWER3 Real-Time PCR System with ChamQ SYBR qPCR Master Mix. Cycle threshold (Ct) values of target gene were normalized to those of housekeeping gene Gapdh an 2^−ΔΔCt^ method was used to calculate relative abundance of gene expression between groups. The primer sequences are displayed in Supplementary Table S1.

### 2.10 HE staining, immunohistochemical staining and immunofluorescence

Colon tissues were fixed by 4% paraformaldehyde, embedded in paraffin, and cut into 4-μm-thick sections. The sections were stained with standard hematoxylin and eosin (H&E) staining and the scoring is presented in Supplementary Table S3. After deparaffinization and rehydration, sections were blocked with 3% hydrogen peroxide. After antigen retrieval, the colon tissue slides were incubated with the primary (Muc2, 1:1000) and secondary antibodies, then 3,3′-diaminobenzidine and hematoxylin were used for immunohistochemical staining (IHC). The sections were photographed by optical microscope and counted with ImageJ software. For immunofluorescence (IF), the colon tissue slides were incubated with the primary (RapGap, 1:500; F4/80, 1:100) and secondary antibodies. After using fluorescein/cy3 tyramide and DAPI to TSA amplification and counterstain the DNA, the Fluorescence microscopy was used to examine all sample preparations.

### 2.11 Flow cytometry

Colonic lamina propria cells were extracted from the research mice in the same manner as previously described (Muller et al., 2014). Colons were isolated, resected, longitudinally opened and cut into pieces. For 30 min at 37°C, intestinal pieces were kept in a digestion mixture containing 5% FBS, 0.5 mg/mL collagenase type IV, 0.5 mg/mL collagenase type I, 0.25 mg/mL DNase. The cell suspensions were centrifuged at 300 g after being filtered through a 70 nm mesh.

After rinsing with 2 mL PBS, the mesenteric lymph-node (MLN) were pulverized with a pestle. The cellular suspension was filtered at 70 nm before being centrifuged at 300 g for 5 min at 4°C. Then supernatant was removed. The single cells in the MLN were obtained after 4 min centrifugation.

Cells were collected and washed in PBS from several experimental sets. Cells were then extracted by centrifugation (300 g for 4 min).

After that, the cells were stained with antibodies and incubated. After incubation, cells were washed in PBS buffer and examined with BD LSRFortessa.

### 2.12 Transcriptome sequencing and bioinformatics analysis

Genome-wide transcriptional sequencing was performed by Majorbio (Shanghai, China). TRIzol^®^ Reagent (Thermo Fisher Scientific, United States) was applied to extract total RNA from colon tissue. Nanodrop2000 (Thermo Fisher Scientific, United States), biowest agarose and 5,300 Bioanalyzer (Agilent, United States) were used for RNA quantification and quality control. The library preparation and sequencing of RNA-seq transcriptome were performed according to Illumina® Stranded (San Diego, CA) at Shanghai Majorbio Bio-pharm Biotechnology Co. Functional enrichment analysis including GO and KEGG were performed to identify which DEGs (Genes with |log2 (foldchange)| ≥1 and adjusted *p* ≤ 0.05 (DESeq2/edgeR/Limma) were considered significantly differentially expressed) were enriched in GO terms and metabolic pathways at Bonferroni-corrected *p*-value ≤0.05 compared with the whole transcriptome background. GO functional enrichment and KEGG pathway analysis were carried out by Goatools^1^ and KOBAS.^2^

### 2.13 UPLC-Q/TOF-MS analysis of metabolites

Chromatographic resolution was successfully implemented on a Acquity HSS T3 column (100 mm × 2.1 mm, 1.8 μm) by utilizing a mobile phase comprising of 0.1% (v/v) formic acid aqueous solution (A) and 0.1% (v/v) formic acid acetonitrile solution (B). The gradient elution procedure was conducted as shown below: 1% B at 0–2 min, 1% → 15% B at 2–3.5 min, 15% → 25% B at 3.5–7.5 min, 25% → 35% B at 7.5–9 min, 35% → 99% B at 9–11.5 min, 99% B at 11.5–17 min, 99% → 1% B at 17–17.1 min, 1% B at 17.1–20.1 min, the column temperature was maintained at 40°C and the autosampler temperature was maintained at 4°C.

The HPLC system was coupled to a Xevo G2-S Q-TOF/MS (Waters) with a Q-Trap^™^ 4000 MS/MS system. Tandem mass spectrometer was manipulated under the positive and negative electrospray ionization (ESI) model. The mass-to-charge ratio range of data acquisition was set at 50–1,000 *m*/*z*. N_2_ was used as the drying gas, and the flow rate was 1.5 L/min, the temperature of N_2_ was 350°C. The capillary voltages of the positive and negative ion modes were set at 2500 V and 2000 V, respectively. And the ion source temperature was set at 120°C, the cone voltage was set at 40 V. The collision energy was set at 20 eV–50 eV. Moreover, in order to obtain the accurate mass of each compound, we used leucine-enkephalin (*m*/*z* 556.2771) as the locked mass solution. All data were acquired and processed by the Waters MassLynx V4.1 software.

### 2.14 Quantitative profiling of BAs

BAs in colonic digesta were measured as previously described (Zeng et al., 2019). The LC-MS/MS analysis was performed on an Agilent 1260 Infinity HPLC system combined with an Agilent 6460 Triple Quadrupole Mass Spectrometry system equipped with an ESI. Chromatographic separations were performed on a BDS Hypersil C18column (50 × 2.1 mm, 2.4 μm, Thermo Scientific) with a Security Guard C18guard column (4 × 2.0 mm, Phenomenex). The acquisition and analysis of data were performed by MassHunter B 05 software.

### 2.15 Statistical analysis

All experiments were repeated at least three times and data were presented as mean ± SD. Statistical differences were analyzed by SPSS 26.0 software (SPSS Inc., Chicago, IL) using one-way ANOVA multiple comparisons and LSD test. *p*-values of less than 0.05 were considered statistically significant.

## 3 Results

### 3.1 LA administration ameliorates DSS-induced murine colitis

In this study, LA intervene exhibited therapeutic effects on colitis mice, as evidenced by reversing body weight loss and colon shortening, along with lower disease activity index (DAI) scores and reduced splenomegaly (Figures 1B,C). Histological examination showed that colitis mice treated with LA dramatically alleviated colon damage and inflammation, including mucosal ulcers, loss of crypt and goblet cells, and epithelial barrier disorders (Figure 1D). Histological scores also revealed that LA could vastly improve the pathological states of the colon (Figure 1D). Additionally, LA was observed to reduce several inflammatory cytokines in the colon, including TNF-α, IL-1β, and IL-6 (Figure 1E). Furthermore, the expressions of Muc-2 and tight junction protein occludin was reduced due to DSS exposure, but after LA intervention, the protein expressions of Muc-2 and occludin were increased (Figure 1F). These data indicated that LA can effectively treat UC.

### 3.2 Intestinal flora is indispensable for LA to ameliorate colitis

The pseudosterile mouse model is often used to investigate the role of intestinal flora in drug treatment (Liu et al., 2020). In our study, we used this model to explore whether LA has an anti-UC effect by acting on the intestinal flora. We found that administration of ABX markedly cleared out fecal microbiota (Supplementary Figure S1). In the ABX + DSS groups, the administration of 3% DSS led to higher body weight loss, colon shortening, spleen index, and DAI score compared to the ABX + Control group. However, these effects were not dramatically alleviated by LA treatment (Figures 2B,C). Hematoxylin and eosin staining of colon sections did not reveal inflamed mucosa in the ABX + Control group. However, in the ABX + DSS and ABX + DSS + LA groups, the colon tissue of mice showed the same mucosal ulcers, loss of crypt, and epithelial barrier disorders (Figure 2D). Consistently, LA did not reduce the level of TNF-α, IL-1β, and IL-6 in the colon (Figure 2E). Mice in the ABX + DSS and ABX + DSS + LA groups showed comparable levels of intestinal barrier damage, and the expression of Muc-2 and occludin did not considerably increase after LA intervention (Figure 2F). These results suggested that intestinal flora is an indispensable factor for LA to ameliorate UC colitis.

### 3.3 LA alters intestinal flora-related metabolites in UC mice, especially bile acids

**Figure 4:**
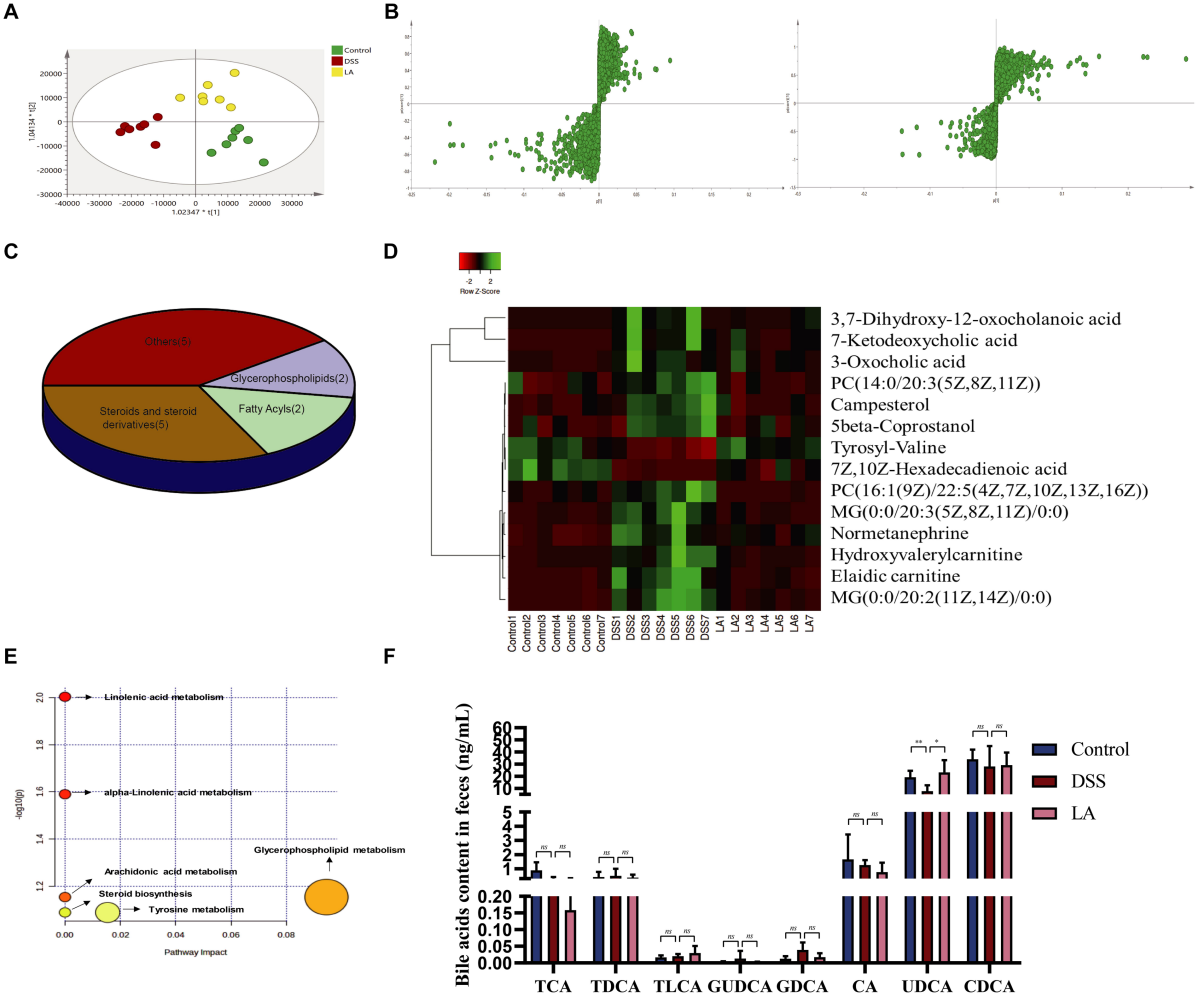
Metabolomics analysis of the effect of LA intervention on fecal metabolites in UC mice. **(A)** PLS-DA plots of the three groups (*n* = 7 mice per group). **(B)** S-plot of OPLS-DA model of Control vs. DSS and DSS vs. LA. **(C)** Classification of 14 differential metabolites. **(D)** Heatmap of differential metabolites altered by LA in UC mice. **(E)** Analysis of metabolic pathways of UC and therapeutic effect of LA. **(F)** Levels of intestinal contents bile acids of cholic acid (CA), chenodeoxycholic acid (CDCA), glycoursodeoxycholic acid (GUDCA), taurocholic acid (TCA), taurochenodeoxycholic acid (TCDCA), glycodeoxycholic acid (GDCA), taurolithocholic acid (TLCA) and ursodeoxycholic acid (UDCA) were detected in three groups (*n* = 6 mice per group). All data were presented as mean ± SD. ^*^p < 0.05 and ^**^p < 0.01.

To further explore the direct correlation between LA and intestinal flora, we analyzed changes in feces metabolites in Control, DSS, and LA mice. Fecal samples from each group were separated using partial least squares discrimination analysis (PLS-DA) score maps (Figure 4A), indicating significant differences in metabolites between the Control, DSS, and LA groups. Representative UPLC-Q/TOF-MS spectra of fecal samples from the three groups are shown in Supplementary Figures S2A–C. We further confirmed that the model was not overfitted and was effective through permutation tests (n = 200), as evidenced by the results displayed in Supplementary Figures S2D,E. The S-plot (Figure 4B; Supplementary Figure S2F) derived from the OPLS-DA model explained most of the variables in the dataset in which the ions farthest away from the origin contributed significantly to the clustering of the two groups and may be regarded as the differential metabolites. Subsequently, differential metabolites were identified with a value of VIP >1 and *p* < 0.05. Therefore, in fecal samples, a variety of different metabolites associated with the UC model were found by comparing the DSS group with the control group, and multiple different metabolites were also identified between the DSS group and the LA group. Further analysis showed that 14 of the differential metabolites between the control and DSS groups were regulated after LA treatment. Notably, five steroids and steroid derivatives (including three bile acids), two fatty acyls, two glycerophospholipids, and five other metabolites were among the 14 differential metabolites (Figure 4C) (Supplementary Table S2). These metabolites were altered in the feces of UC mice and returned to normal after LA intervention (Figure 4D). Enrichment analysis revealed that glycerophospholipid metabolism, linoleic/alpha-linoleic acid metabolism, tyrosine metabolism, and steroid biosynthesis were critically involved (Figure 4E). It can be seen that steroids and their derivatives are a class of metabolites that have the most significant effect on the intervention of LA in UC, and LA markedly affects the synthesis of steroids and their derivatives while treating UC. Primary bile acid is a kind of steroid synthesized by cholesterol in the liver (Mizuochi et al., 2023). According to research, it is converted into secondary bile acid under the action of intestinal flora and acts as a signal transduction molecule to affect the sensitivity to intestinal inflammation. At the same time, abnormal signal transduction of bile acid may also lead to the imbalance of UC immune response.

Considering the bile acid metabolism may be involved in LA treatment of DSS predicted by UPLC/Q-TOF-MS, we further conducted targeted the contents of CA, CDCA, glycoursodeoxycholic acid (GUDCA), taurocholic acid (TCA), taurochenodeoxycholic acid (TCDCA), glycodeoxycholic acid (GDCA), taurolithocholic acid (TLCA) and UDCA were determination in feces samples from mice. Quantitative analysis of bile acid species indicated that GDCA and GUDCA exhibited an increasing trend in the DSS group, although statistical significance was not reached, and the concentrations of both were markedly low in the mice’s gut. The contents of TCA, CA, and UDCA all showed a decreasing trend in the DSS group, but only the contents of UDCA increased significantly after LA intervention (Figure 4F). These data indicated that UDCA was most upregulated in the bile acid of DSS treated with LA. Therefore, we decided to use UDCA directly to interfere with colitis mice in next study, aimed to explore the therapeutic effects of UDCA on UC mice.

### 3.4 LA suppresses disease development in UC mice by modulating immune responses

**Figure 5:**
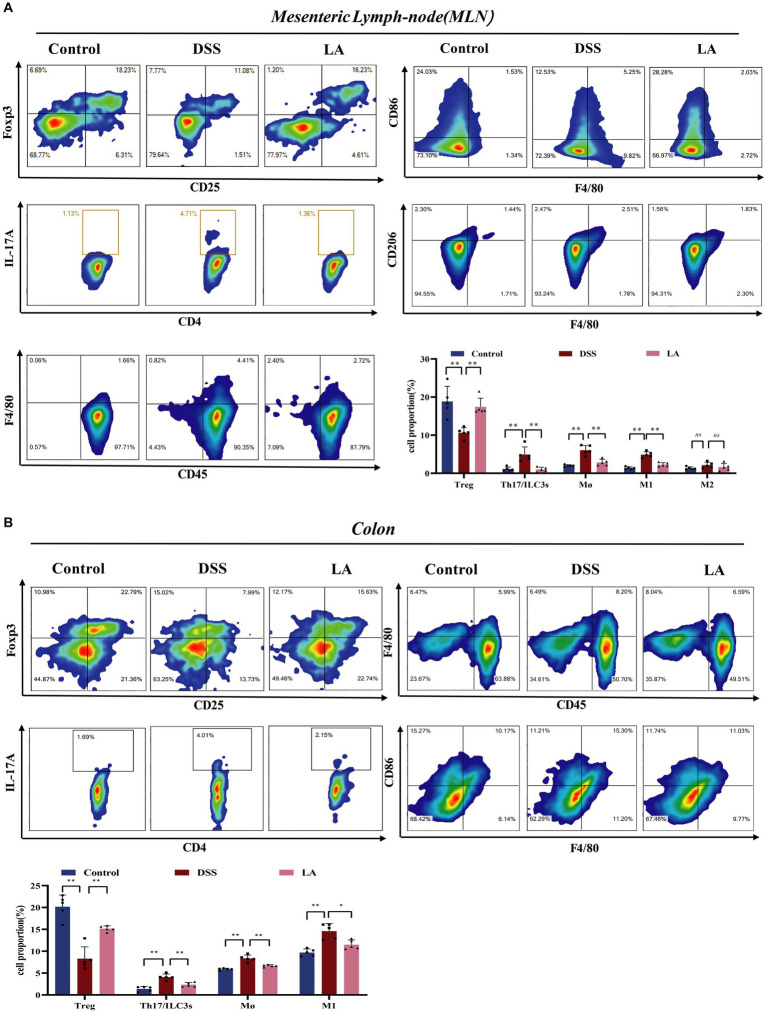
LA maintained the immune homeostasis in DSS-induced mice. **(A)** In mesenteric lymph-node (MLN): CD4^+^CD25^+^Foxp3^+^ Treg cells; CD4^+^IL-17a^+^ Th17 cells; CD45^+^F4/80^+^ macrophages; CD45^+^F4/80^+^CD86^+^ M1 macrophages; CD45^+^F4/80^+^CD206^+^ M2 macrophages. **(B)** In colon: CD4^+^CD25^+^Foxp3^+^ Treg cells; CD4^+^IL-17a^+^ Th17 cells; CD45^+^F4/80^+^ macrophages; CD45^+^F4/80^+^CD86^+^ M1 macrophages. All data were presented as mean ± SD (*n* = 5 mice per group). ^*^p < 0.05, ^**^p < 0.01, and ^ns^*p* > 0.05.

Inflammation of the gut is influenced by intracellular immunity. Immune cells respond to acute inflammatory reactions by clearing out bacteria and pathogens, protecting the health of the gut (Neurath, 2014; Tian et al., 2022; Zhao et al., 2023). Increased numbers of Th17 and ILC3s (CD4^+^IL-17^+^) cells and decreased numbers of Treg (CD4^+^CD25^+^Foxp3^+^) cells were detected in the peripheral blood of patients suffering from UC, and the imbalance of these cell types contributes to inflammation in this disease (Cinelli et al., 2020). As presented in Figures 5A,B, the number of Th17 and ILC3s cells increased, and the number of Treg cells decreased in the DSS group compared with the Control group. Administration of LA decreased Th17 and ILC3s cells and increased Treg cells. During the active phase of UC, the population of intestinal macrophages, derived from circulating monocytes, increases. Activated macrophages produce excessive inflammatory cytokines during the process of ulcerative colitis. Flow cytometry showed that the total macrophage (CD45^+^F4/80^+^) and M1 macrophage (CD45^+^F4/80^+^CD86^+^) numbers increased in the DSS group but decreased after LA intervention. Interestingly, we found no significant change in the number of M2 macrophages (CD45^+^F4/80^+^CD206^+^) in the MLN and colon of the three groups of mice. Overall, these findings indicated that LA could restore immune disorders in UC mice.

### 3.5 UDCA administration also ameliorates DSS-induced murine colitis

The experimental schedule for the mouse study is depicted in Figure 3A. Compared to the Control group, the DSS group displayed weight loss, severe DAI score, spleen index, and colon shortening, which were partially ameliorated after UDCA intervention (Figures 3B–D). Histological examination revealed that the DSS mice exhibited several characteristic changes, including mucosal ulcers, loss of crypt and goblet cells, and epithelial barrier disorders. Interestingly, UDCA-M, UDCA-H, and SASP radically ameliorated these changes, while the administration of UDCA-L also had a protective effect on colon morphology, albeit not as significant as that of UDCA-M and UDCA-H (Figures 3E,F). Furthermore, the expression of inflammatory factors, including TNF-α, IL-1β, and IL-6, remarkably increased in the DSS group compared to the Control group. Fortunately, these increases were attenuated by UDCA-M, UDCA-H, and Sulfasalazine (SASP) (Figure 3G). Consistently, the expression of Muc-2 and occludin decreased in the DSS group compared with the Control group, whereas UDCA-M, UDCA-H, and SASP up-regulated their expression (Figure 3H). However, UDCA-L did not improve the expression of these proteins compared to the DSS group. Overall, the results suggest that UDCA-M and UDCA-H have significant therapeutic potential for UC, while UDCA-L may not be as effective. These findings support that bile acids, particularly UDCA, are effector molecules for LA to exert its therapeutic effect on UC.

### 3.6 UDCA also modulated mesenteric lymph nodes and colon immune responses in UC mice

**Figure 6:**
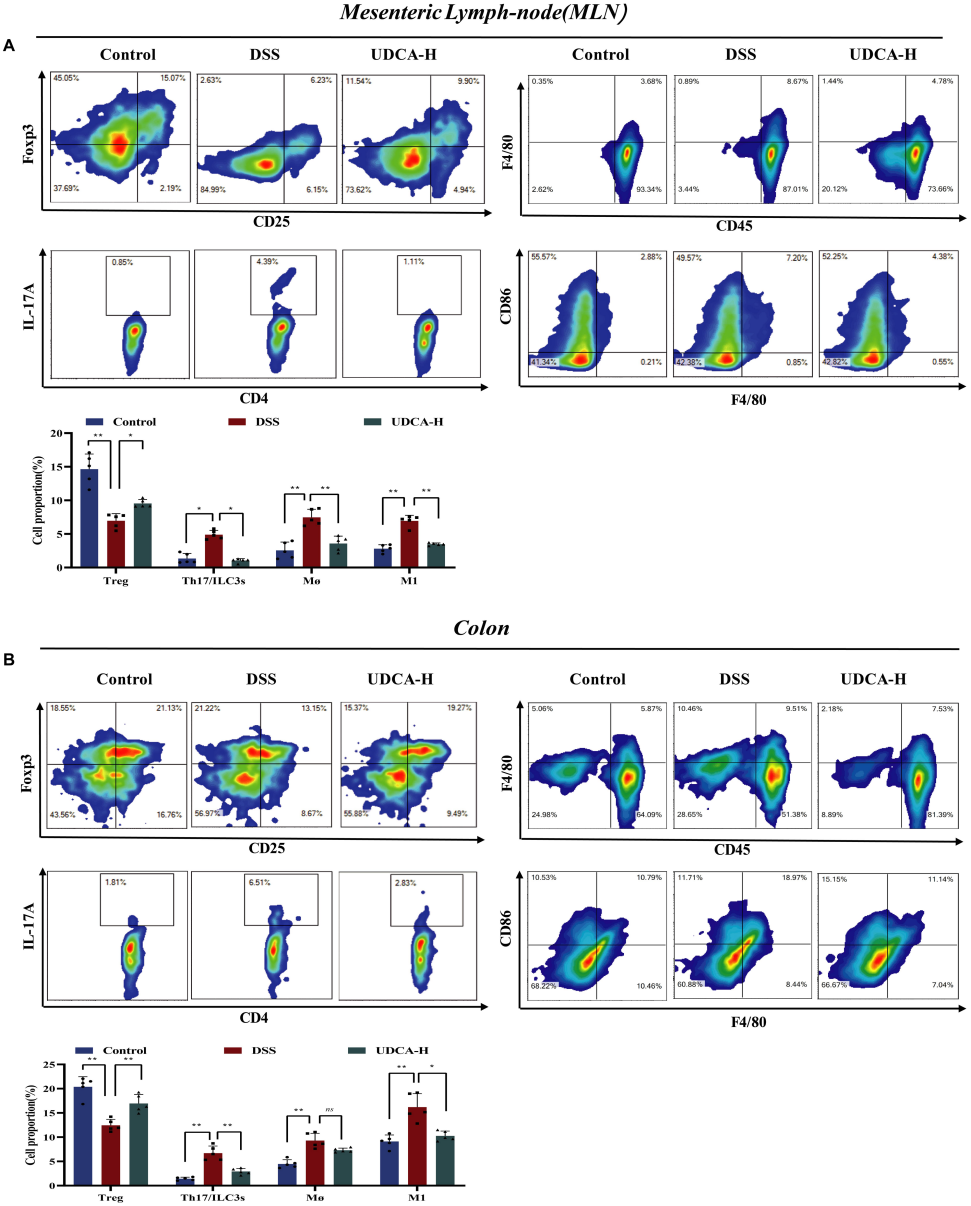
UDCA maintained the immune homeostasis in DSS-induced mice. **(A)** In mesenteric lymph-node (MLN): CD4^+^CD25^+^Foxp3^+^ Treg cells; CD4^+^IL-17a^+^ Th17 cells; CD45^+^CD103^+^ Dendric cells; CD45^+^NK1.1^+^ Natural kill cells; CD45^+^F4/80^+^ macrophages; CD45^+^F4/80^+^CD86^+^ M1 macrophages. **(B)** In colon: CD4^+^CD25^+^Foxp3^+^ Treg cells; CD4^+^IL-17a^+^ Th17 cells; CD45^+^CD103^+^ Dendric cells; CD45^+^NK1.1^+^ Natural kill cells; CD45^+^F4/80^+^ macrophages; CD45^+^F4/80^+^CD86^+^ M1 macrophages. All data were presented as mean ± SD (*n* = 5 mice per group). ^*^p < 0.05, ^**^p < 0.01, and ^ns^*p* > 0.05.

We conducted a study to explore whether UDCA-H, like LA, can regulate mesenteric lymph node and colon immune cells in UC mice. As shown in Figures 6A,B, our findings were consistent with previous research, demonstrating that the percentage of Th17 and ILC3s cells increased, while the percentage of Treg cells decreased in the DSS group compared with the Control group. Administration of UDCA decreased Th17 and ILC3s cells and increased Treg cells. Furthermore, the percentage of total macrophage and M1 macrophage cells in MLN and colon were considerably decreased in the presence of UDCA. These results suggest that UDCA is involved in the immune response of MLN and colon, which affects gut barrier function and disease progression in UC mice. Overall, our findings demonstrate the significant therapeutic effect of UDCA-H on the immune cell response and provide insight into the potential mechanism underlying the protective effect of UDCA in UC.

### 3.7 UDCA directly promoted Treg polarization and suppressed M1 macrophage polarization

**Figure 7:**
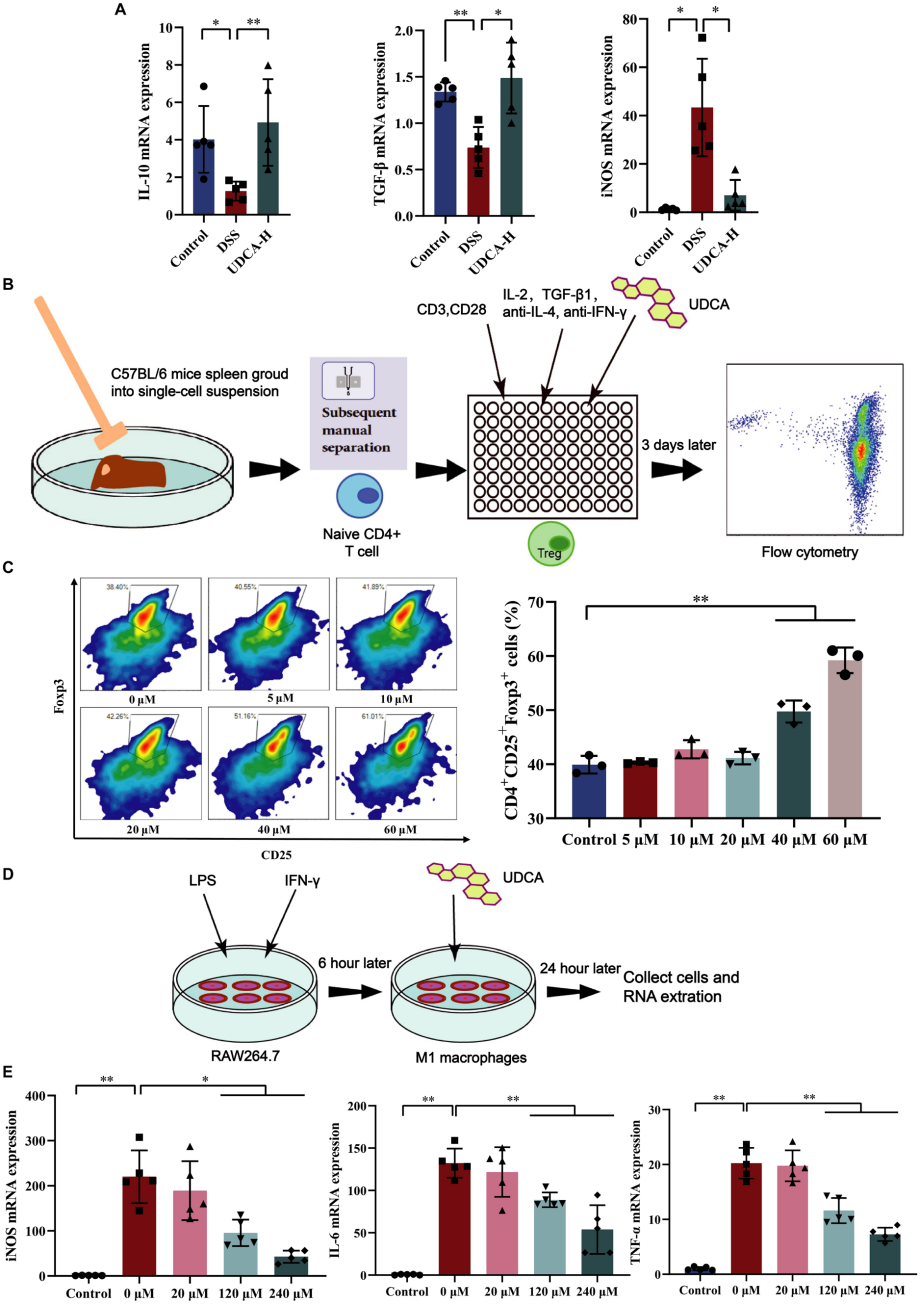
UDCA induced differentiation of Tregs and inhibited differentiation of M1 macrophages *in vitro*. **(A)** The mRNA expression of IL-10, TGF-β, iNOS in colon (*n* = 5 mice per group). **(B)** Tregs differentiation process *in vitro*. **(C)** Effect of UDCA on the differentiation of Treg cells (*n* = 3 per group). **(D)** M1 macrophages differentiation process *in vitro*. **(E)** Effect of UDCA on the differentiation and related cytokines of M1 macrophages (*n* = 3 per group). All data were presented as mean ± SD. ^*^p < 0.05 and ^**^p < 0.01.

Alleviating inflammation by modulating immune cells is a commonly used immunotherapy for UC clinical treatment. Our results showed that UDCA can promote IL-10 and TGF-β expression and inhibit iNOS expression (a marker of M1 macrophage polarization) in UC mice (Figure 7A). However, the direct regulatory effect of UDCA on Tregs and M1 macrophages has not been thoroughly examined. To investigate this, *in vitro* experiments were conducted where Treg cells and M1 macrophages were stimulated and differentiated, and UDCA was used as an intervention (Figures 7B,D). Results showed that UDCA was found to substantially affect the differentiation of Treg cells, promoting Treg cell differentiation as shown by increased expression of Foxp3 (Figure 7C). When M1 macrophages were polarized with LPS and IFN-γ, UDCA treatment for 24 h reduced iNOS levels in a dose-dependent manner (Figure 7E). Additionally, the mRNA expression of TNF-α and IL-6, cytokines secreted by M1 macrophages with pro-inflammatory effects, were decreased after UDCA treatment (Figure 7E). These results suggest that UDCA can increase the secretion of anti-inflammatory factors by promoting Treg cell differentiation and inhibits proinflammatory cytokine secretion by inhibiting the polarization of M1 macrophages. Therefore, UDCA may have a direct regulatory effect on Tregs and M1 macrophages, contributing to its therapeutic effect in UC.

### 3.8 LA regulates UDCA improved UC mice by RapGap/PI3K-AKT/NF-κB signal pathway

**Figure 8:**
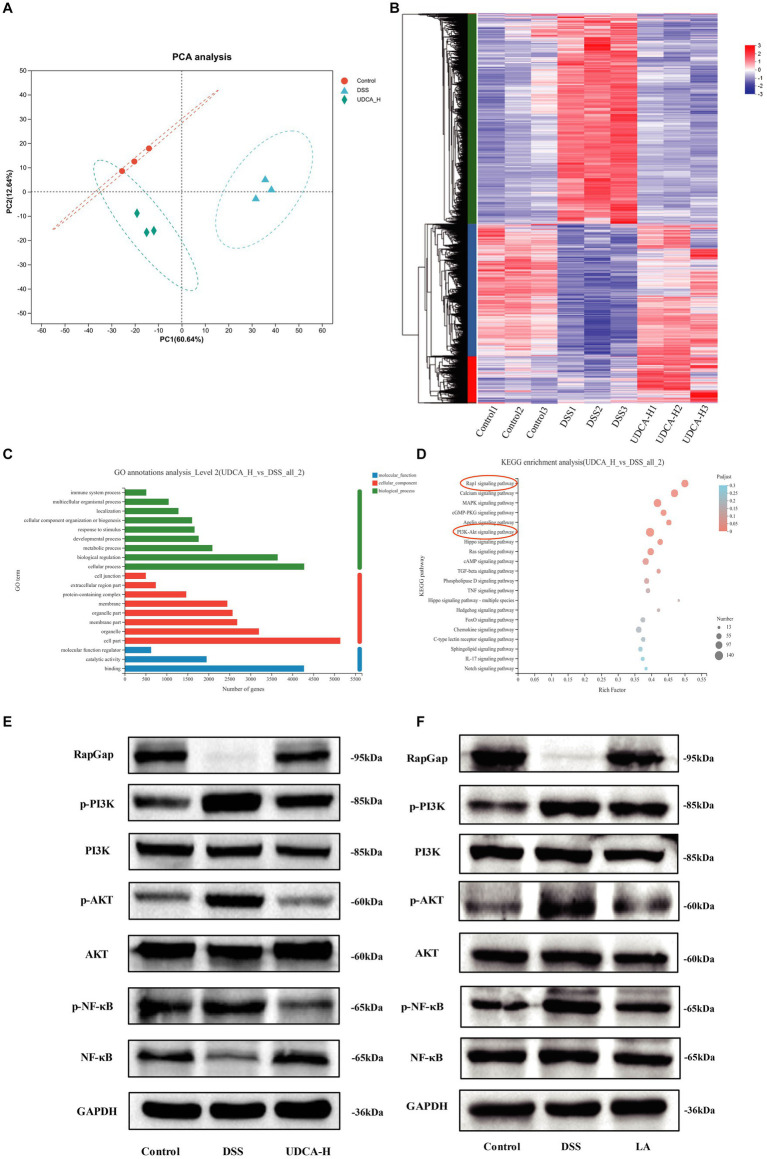
Transcriptomics analysis of the potential mechanism of UDCA treatment in UC mice. **(A)** Principal component analysis (PCA) between the Control, DSS and UDCA. **(B)** Heatmap of distinctly dysregulated mRNAs in three groups (Control, DSS, UDCA) are identified from transcriptome sequencing by hierarchical clustering. High and low expression levels are indicated in red and blue, respectively. **(C)** GO annotations analysis of distinctly dysregulated in DSS and UDCA groups identified from transcriptome sequencing. **(D)** KEGG pathway analysis of distinctly dysregulated pathways in DSS and UDCA groups identified from transcriptome sequencing. **(E)** RapGap/PI3K-AKT/NF-κB expression in three groups (Control, DSS, UDCA) is detected by western-blot. **(F)** RapGap/PI3K-AKT/NF-κB expression in three groups (Control, DSS, LA) is detected by western-blot.

To explore the possible mechanisms accounting for the role of UDCA in the development of UC, we performed transcriptome sequencing with Control, DSS and UDCA mice colon. The PCA analysis showed that UDCA-treated mice gradually returned to a gene expression profile similar to the Control group (Figure 8A). This is supported by the hierarchical clustering analysis which showed that UDCA-treated mice have a gene expression pattern more similar to the Control group than the DSS group (Figure 8B). Veen diagram showed that among the 16,258 genes detected, 12,442 genes are shared by the three groups (Supplementary Figure S3A). We identified 5,240 upregulated genes and 5,128 downregulated genes in DSS versus Control, 4,675 upregulated genes and 4,858 downregulated genes in UDCA versus DSS (Supplementary Figure S3B). Furthermore, GO function annotation analysis indicated that the differentially expressed genes between UDCA and DSS groups were mainly involved in immune system processes, biological regulation, and cellular processes (Figure 8C). GO annotations analysis results of distinctly dysregulated in Control and DSS groups showed in Supplementary Figure S3C.

The transcriptome sequencing and KEGG pathway analysis demonstrated that UDCA treatment enriched the Rap1 pathway and PI3K-AKT pathway, which is consistent with the KEGG pathway enrichment results obtained in the DSS vs. Control groups (Figure 8D; Supplementary Figure S3D). The Rap1 signaling pathway diagram indicates that the PI3K-AKT signaling pathway is downstream of the Rap1 signaling pathway (Supplementary Figure S3E). Since the induction of iNOS is mediated via NF-kB, and iNOS mRNA expression was lowered by UDCA (Belkaid and Harrison, 2017), we indicated UDCA can also regulate nuclear factor kappa-B (NF-κB) pathway in UC mice. Based on the differential expression of genes involved in these pathways, it was hypothesized that UDCA could modulate the DSS phenotype by regulating the RapGap/PI3K-AKT/NF-κB pathway. The western blot analysis validated that in DSS-treated mice, RapGap was downregulated, and phosphor-Phosphatidylinositol-3 kinase (p-PI3K), phosphor-protein kinase B (p-AKT), and phosphor-nuclear factor kappa-B (p-NF-κB) were upregulated. Following UDCA intervention, this situation was partially reversed (Figure 8E), and the LA intervention also showed the same effect (Figure 8F). The protein expression was showed in Supplementary Figure S3F. Thus, these results indicate that LA regulates UDCA could improve colitis by modulating the RapGap/PI3K-AKT/NF-κB signaling pathway in UC mice.

### 3.9 RapGap is required for UDCA-regulated macrophages

**Figure 9:**
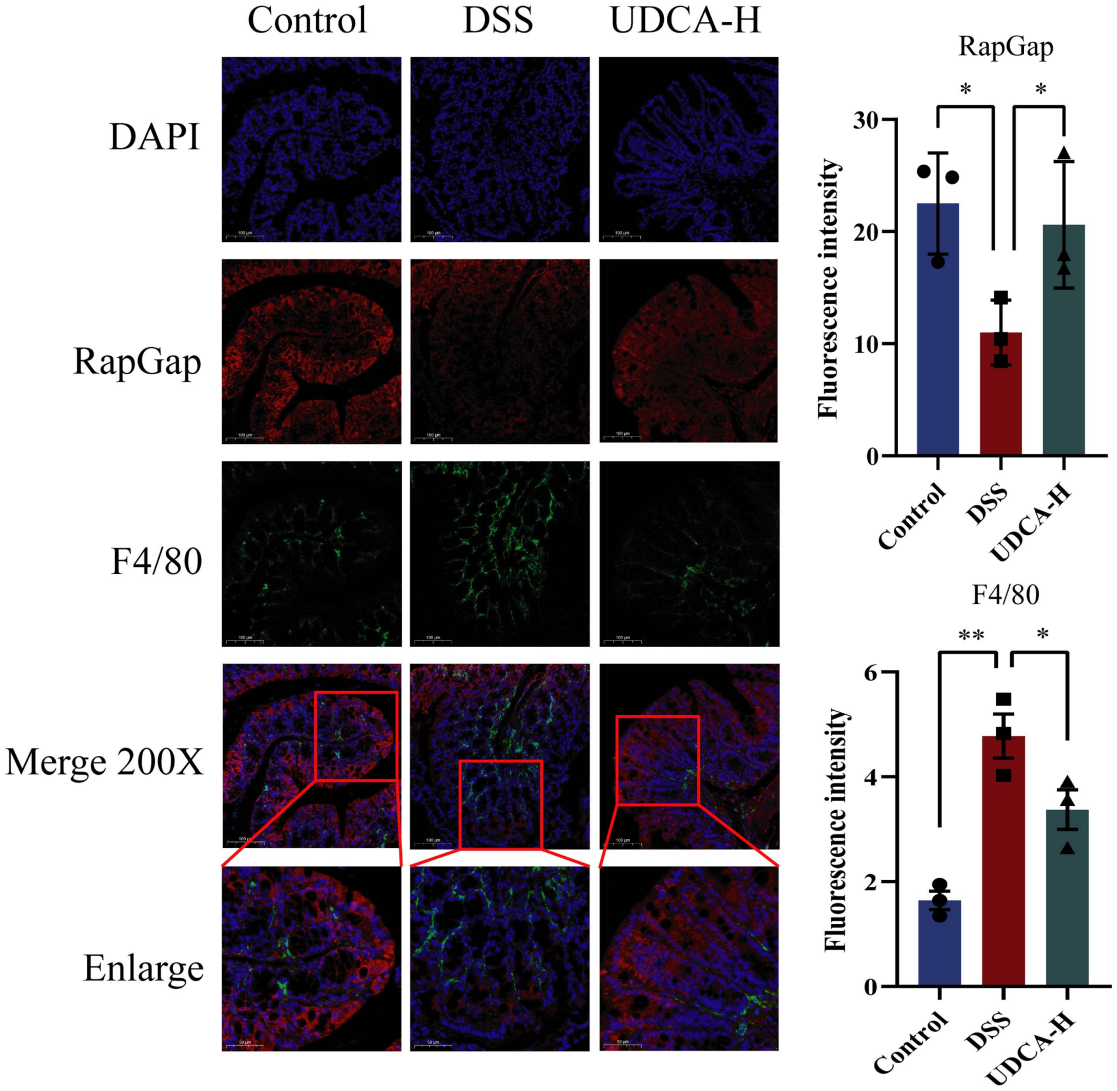
Expression changes of RapGap and F4/80 in colon tissue of the same mouse in different groups. All data were presented as mean ± SD (*n* = 3 mice per group). ^*^p < 0.05 and ^**^p < 0.01.

To explore the relationship between the immune system and the RapGap/PI3K-AKT/NF-κB signaling pathway in UDCA-treated UC mice, we used immunofluorescence to investigate the correlation between RapGap and macrophages. Our results showed a significant reduction in RapGap expression and an increase in F4/80 expression in the colon tissue of the DSS group compared to the Control group. However, after UDCA intervention, RapGap expression increased, and F4/80 expression decreased. These findings suggest that changes in RapGap expression are associated with changes in F4/80 expression, indicating a significant negative correlation between the Rap1 signaling pathway and macrophages in colitis mice (Figure 9). These findings suggest that during the alleviation process of UC, both the Rap1 signaling pathway and macrophages are regulated by UDCA, indicating a potential correlation between the two.

## 4 Discussion

Intestinal flora and metabolites play an indispensable role in inducing the development of a mature intestinal immune system, as indicated by previous studies (Shen et al., 2014; Li Q. et al., 2022; Shan et al., 2022; Liang et al., 2023; Wu et al., 2023). The host and the microorganism form an intestinal mucosal steady state to ensure the normal function of the intestine (Round and Mazmanian, 2009). Immune disorders caused by intestinal flora dysbiosis exert extensive effects on the pathogenesis of UC (Ananthakrishnan, 2015; Shan et al., 2022). Thus, investigating the contributions of microorganisms and metabolites to the pathogenesis of UC holds promise for novel preventive and therapeutic approaches. In this study, we comprehensively examine the effects of LA and its metabolites on gut inflammation recovery in a DSS-induced colitis mouse model. The observed alleviation of inflammation is primarily attributed to the regulation of intestinal metabolites, immune microenvironment, and intestinal flora.

Our findings demonstrate the efficacy of LA in ameliorating DSS-induced colitis symptoms, evidenced by reduced colon length, weight loss, DAI score, and mucosal loss, corroborating prior studies (Park et al., 2018). Numerous studies have established that germ-free and antibiotic-treated mice are more susceptible to epithelial damage and severe hemorrhage in DSS-induced colitis (Hernandez-Chirlaque et al., 2016). In our current investigation, we observed that the protective effect of LA was diminished in mice treated with ABX, suggesting that LA’s anti-inflammatory effects are likely mediated indirectly through intestinal flora regulation. The intestinal epithelial barrier is essential for protecting against inflammation and harmful invasion, with goblet cells playing a critical role in this protection through the secretion of various mucus types that strengthen the colon epithelial barrier (Chen et al., 2018; Luo et al., 2022). Previous research has suggested that the reactive oxygen species (ROS) produced by prolonged inflammation could accelerate the reduction in intestinal permeability. To evaluate the effect of LA on colon intestinal epithelial barrier, we utilized H&E staining, western blotting, and immunohistochemistry staining, revealing that DSS-induced histopathological changes in the epithelial barrier, such as enterocyte loss, inflammatory infiltration, and crypt structure disruption, were mitigated following LA treatment. Moreover, LA demonstrated the ability to alleviate the reduction in intestinal permeability resulting from intestinal damage.

Additionally, we unveil LA’s capacity to modulate steroid biosynthesis and promote BA synthesis. Existing literature supports the anti-inflammatory effects of BAs in murine colitis models, with LCA and DCA reported to alleviate inflammation and exert immuno-modulating effects, respectively, both *in vitro* and *in vivo* (Guo et al., 2016; Ward et al., 2017). HDCA, on the other hand, has been found to suppress intestinal epithelial cell proliferation and alter gut bacteria and BA profiles, with HDCA analogs providing protection against TNBS-induced colitis (Marino et al., 2020; Song et al., 2020). UDCA, an important BA, has been approved as a standard treatment for primary biliary cholangitis, a previous study showed that UDCA treatment improves dysplasia in patients with IBD and reduces the risk of adenoma recurrence (Carey et al., 2015; Pearson et al., 2019). In our study, we confirmed UDCA ameliorated DSS-induced colitis and accelerated mucosal repair.

Notably, several mucosal immune cells, including T cells and macrophages, can be activated by bile acids (BAs), leading to their anti-inflammatory effects (Jia et al., 2018). Hence, we investigated intestinal immune responses in colitis models and observed that the intestinal immune response was diminished following LA and UDCA intervention. Disruption of the Treg/Th17 balance is considered a crucial factor in inflammatory bowel diseases (IBDs). Treg cells play a pivotal role in suppressing tissue inflammation, dependent on the effectors TGF-β and IL-10, while Th17 cells are pro-inflammatory, primarily secreting IL-17A and closely linked to IBD severity (Britton et al., 2019; Yan et al., 2020). Our data supported the notion that the corrected Treg/Th17 immune balance is associated with the protective role of LA and UDCA in murine colitis. Further investigations revealed that UDCA increased the expression of IL-10 and TGF-β in colitis and promoted Treg cell differentiation *in vitro*. *In vitro* experiments further indicated that UDCA directly inhibits the polarization of M1 macrophages and reduces the expression of inflammatory cytokines, such as TNF-α, IL-1β and IL-6. Additionally, LA and UDCA suppressed the elevation of pro-inflammatory M1 macrophages, rather than inducing anti-inflammatory M2 macrophage subsets under colitis conditions, suggesting that LA and UDCA may ameliorate colitis by inhibiting intestinal M1 macrophages. In conclusion, our study unravels the beneficial roles of LA and UDCA in conferring resistance to DSS-induced damage through the regulation of immune responses. Furthermore, we used transcriptomics to analyze the signaling pathways through which UDCA regulates immune cells, and found that UDCA can greatly affect the Rap1 signaling pathway and the PI3K-AKT signaling pathway.

Rap1, a small GTP-binding protein, undergoes cycling between the GTP-bound and GDP-bound states (Caron, 2003; Gloerich and Bos, 2011). The Rap1 signaling pathway has been recognized as an essential inflammatory protective pathway in inflammatory diseases (Yang et al., 2023). RapGap serves as the GTP enzyme activating protein for Rap1, facilitating the hydrolysis of GTP to GDP, thereby inactivating Rap1 and inhibiting the Rap1 pathway’s activity. Meanwhile, the PI3K-AKT pathway, a classical intracellular signaling cascade responsive to extracellular signals, regulates metabolism, proliferation, cell survival, growth, and angiogenesis, and is activated by the Rap1 signaling pathway (Burke, 2018). Aberrant activation of the PI3K-AKT pathway in the pathogenesis of UC leads to cytokine expressions and oxidative stress, disrupting the immune system balance (El-Mihi et al., 2017; Nunes-Santos et al., 2019). Furthermore, Rap1 has been shown to activate the NF-kB signaling pathway (Ling et al., 2023). In our investigation, we made the noteworthy observation that both LA and UDCA upregulated the expression of RapGap protein while inhibiting the expression of PI3K, AKT, and NF-κB proteins. This strongly suggests that LA and UDCA exert their ameliorative effects on colitis through the RapGap/PI3K-AKT/NF-κB signaling pathway. Moreover, we found a significant negative correlation between RapGap and macrophages, indicating that LA and UDCA may modulate macrophages via the RapGap/PI3K-AKT/NF-κB pathway to effectively treat UC.

## 5 Conclusion

In summary, we propose that the role of LA in alleviating UC may be linked to its upregulation of UDCA. Both LA and its metabolite, UDCA, treat UC by activating the RapGap/PI3K-AKT/NF-κB signaling pathway and modulating Treg cells and M1 macrophages. Our study underscores the critical role of microbial metabolites and gut immune cells, particularly UDCA, M1 macrophages and Tregs, in regulating gut inflammation and offers valuable insights for the development of innovative UC therapeutic strategies based on the modulation of bile acid profiles and immune cells-targeted interventions.

## Data availability statement

The original contributions presented in the study are publicly available. This data can be found here: PRJNA1033699.

## Ethics statement

The animal study was supervised and approved by the Animal Ethics Committee of Guangzhou University of Chinese Medicine (Approval No. ZYD-2022-024). The study was conducted in accordance with the local legislation and institutional requirements.

## Author contributions

SD: Conceptualization, Formal analysis, Writing – original draft. CP: Conceptualization, Formal analysis, Writing – original draft. KC: Conceptualization, Formal analysis, Writing – original draft. WH: Data curation, Formal analysis, Methodology, Writing – review & editing. XX: Formal analysis, Investigation, Writing – review & editing. XZ: Data curation, Methodology, Writing – review & editing. RL: Data curation, Formal analysis, Writing – review & editing. YC: Formal analysis, Investigation, Writing – review & editing. ZX: Methodology, Supervision, Writing – review & editing. PL: Methodology, Supervision, Writing – review & editing. QL: Formal analysis, Methodology, Writing – review & editing.

## Footnotes

^1^ https://github.com/tanghaibao/Goatools

^2^ http://kobas.cbi.pku.edu.cn/home.do

## Supplementary material

Supplementary Figure S1 Agarose gel electrophoresis to detect the effectiveness of the antibiotic treatment.

Supplementary FIGURE S2 Non-target metabolomics analysis of metabolites in feces. UPLC-Q/TOF-MS spectra of fecal samples of mice from **(A)** Control, **(B)** DSS and **(C)** LA groups. Permutations tests (*n* = 200) of Control vs DSS **(D)** and DSS vs LA **(E)**. **(F)** OPLS-DA score plots of Control vs DSS and DSS vs LA.

Supplementary FIGURE S3 Transcriptomics analysis of the potential mechanism of UDCA treatment in UC mice. **(A)** Veen diagram analysis. A volcano plot illustrating differentially regulated gene expression between the Control, DSS and UDCA. **(B)** Genes upregulated and downregulated are shown red and green, respectively. **(C)** GO annotations analysis of distinctly dysregulated in Control and DSS groups identified from transcriptome sequencing. **(D)** Pathway analysis of distinctly dysregulated pathways in Control and DSS groups identified from transcriptome sequencing. **(E)** Diagram of the RAP1 signaling pathway. **(F)** RapGap/PI3K-AKT/NF-κB expression in three groups (Control, DSS, UDCA) is detected by Western-blot. All data were presented as mean±SEM (*n* = 3 mice per group). **P* < 0.05, ***P* < 0.01, ns *P* > 0.05.
