# Supplementary figures and images for: Lactobacillus acidophilus and its metabolite ursodeoxycholic acid ameliorate ulcerative colitis by promoting Treg differentiation and inhibiting M1 macrophage polarization
Source: Front Microbiol. 2024 Jan 16;15:1302998. doi: 10.3389/fmicb.2024.1302998 (PMC10825044; doi:10.3389/fmicb.2024.1302998)

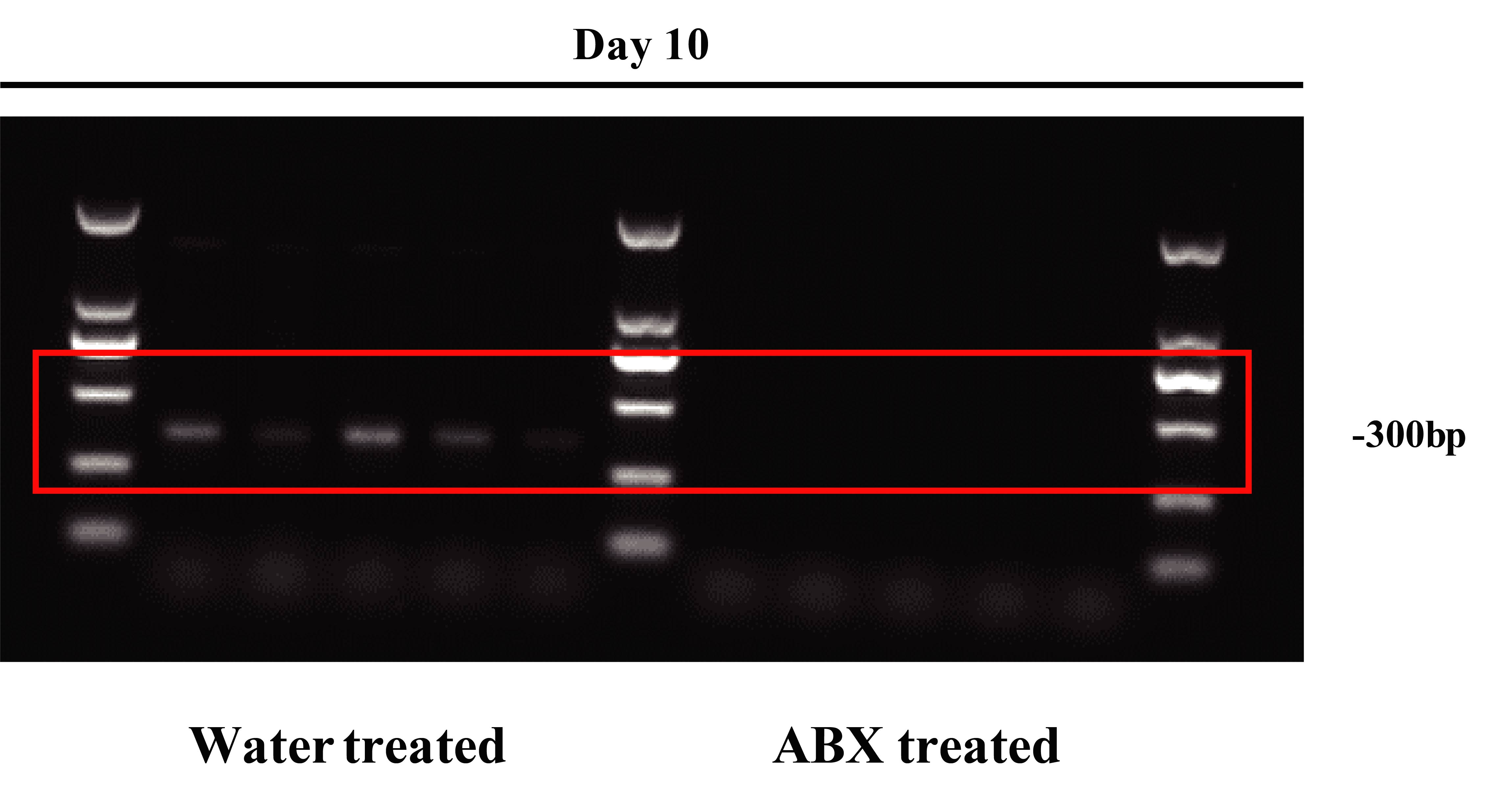

Supplement: Supplementary Figure S1 — Agarose gel electrophoresis to detect the effectiveness of the antibiotic treatment. [file Image_1.JPEG]

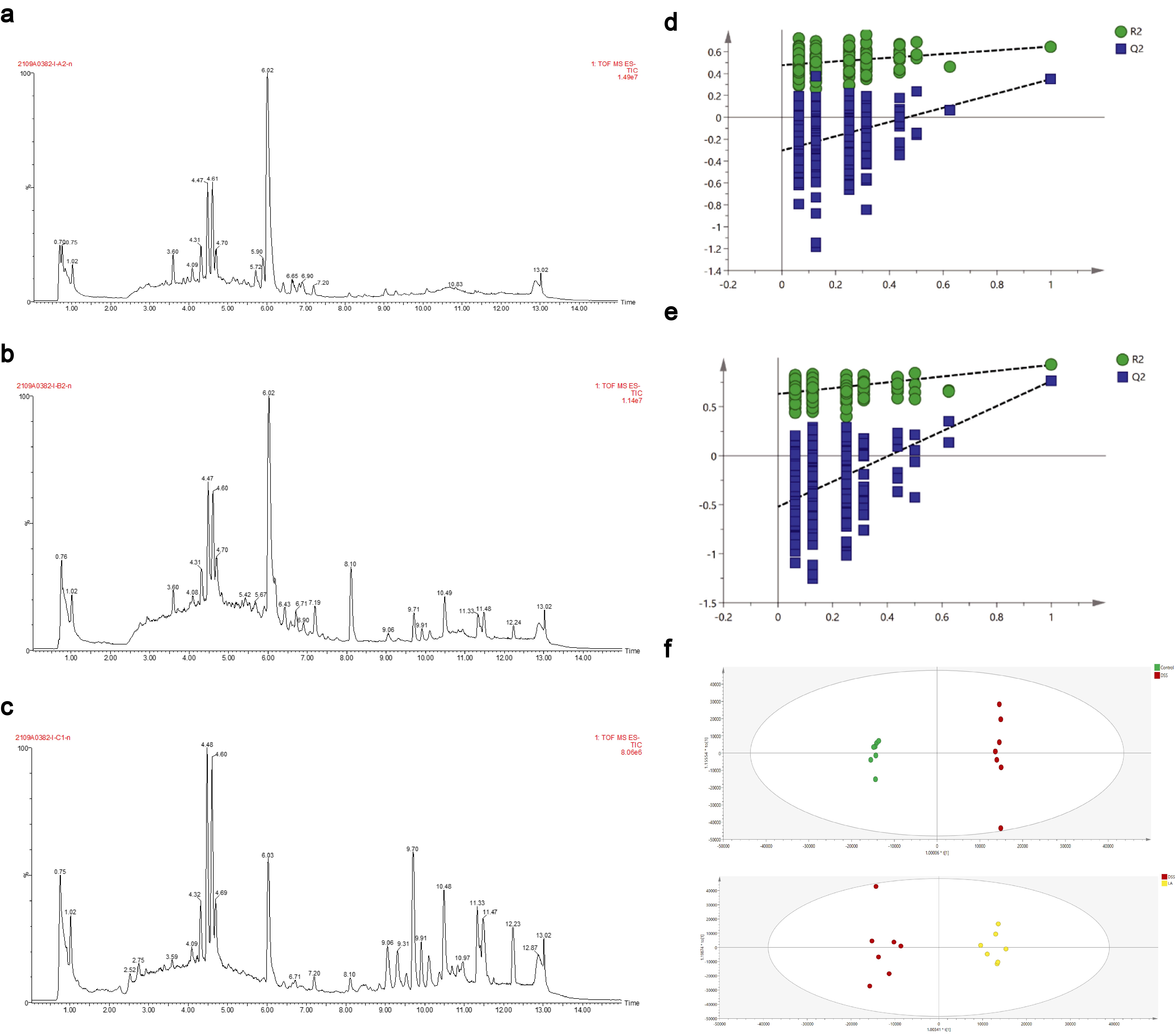

Supplement: Supplementary FIGURE S2 — Non-target metabolomics analysis of metabolites in feces. UPLC-Q/TOF-MS spectra of fecal samples of mice from (A) Control, (B) DSS and (C) LA groups. Permutations tests (n = 200) of Control vs DSS (D) and DSS vs LA (E). (F) OPLS-DA score plots of Control vs DSS and DSS vs LA. [file Image_2.JPEG]

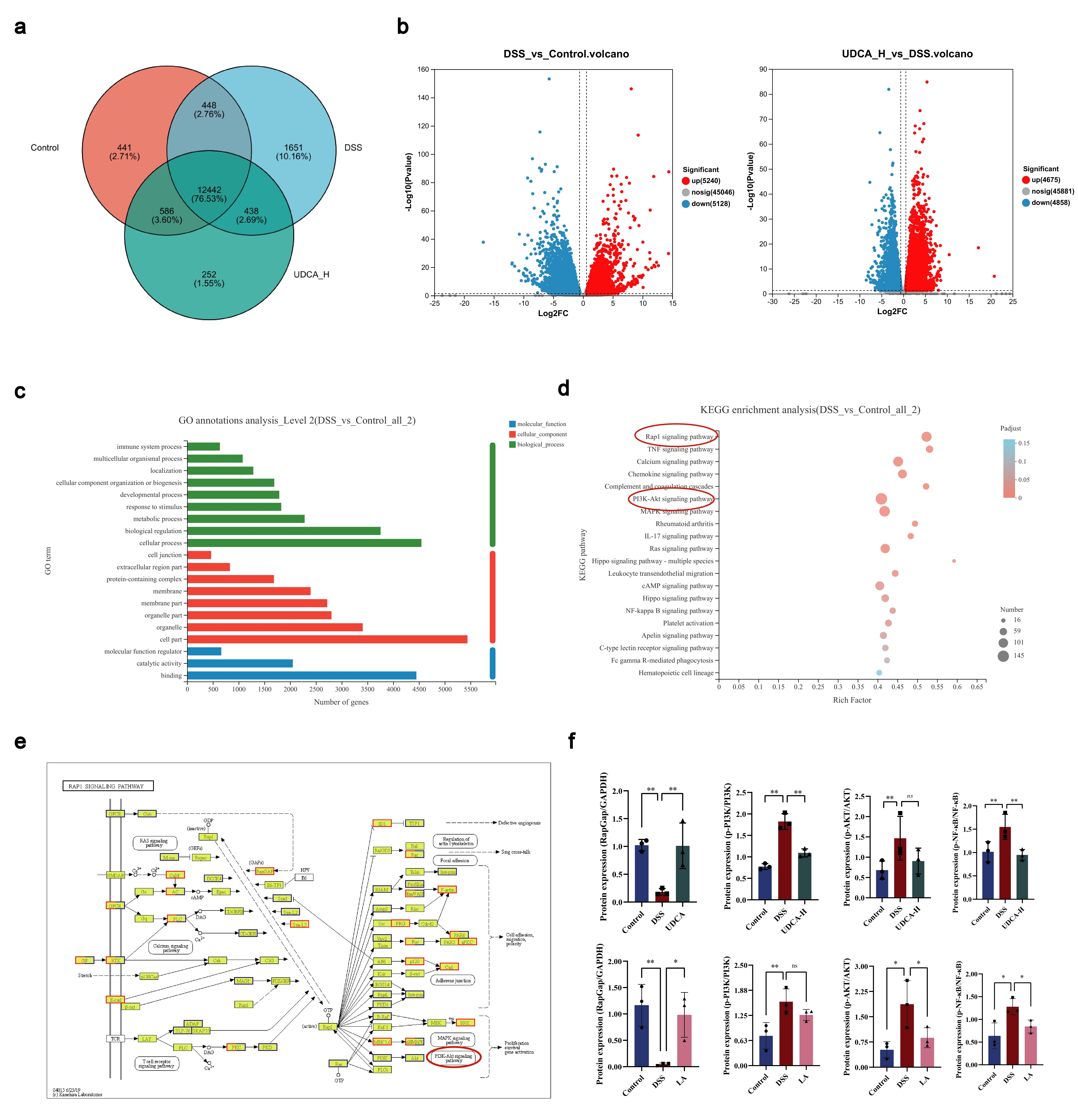

Supplement: Supplementary FIGURE S3 — Transcriptomics analysis of the potential mechanism of UDCA treatment in UC mice. (A) Veen diagram analysis. A volcano plot illustrating differentially regulated gene expression between the Control, DSS and UDCA. (B) Genes upregulated and downregulated are shown red and green, respectively. (C) GO annotations analysis of distinctly dysregulated in Control and DSS groups identified from transcriptome sequencing. (D) Pathway analysis of distinctly dysregulated pathways in Control and DSS groups identified from transcriptome sequencing. (E) Diagram of the RAP1 signaling pathway. (F) RapGap/PI3K-AKT/NF-κB expression in three groups (Control, DSS, UDCA) is detected by Western-blot. All data were presented as mean±SEM (n = 3 mice per group). *P < 0.05, **P < 0.01, ns P > 0.05. [file Image_3.JPEG]
