# Supplementary material for: Lactobacillus acidophilus and its metabolite ursodeoxycholic acid ameliorate ulcerative colitis by promoting Treg differentiation and inhibiting M1 macrophage polarization
Source: Front Microbiol. 2024 Jan 16;15:1302998. doi: 10.3389/fmicb.2024.1302998 (PMC10825044; doi:10.3389/fmicb.2024.1302998)
Supplement: Supplementary file 4 [file Table_1.DOCX]

**Table S1 Primer sequences for real-time PCR used in the study.**

| Target genes | Primer sequence | |
| --- | --- | --- |
|  | Forward 5'-3' | Reverse 5'-3' |
| IL-1β | TTCAGGCAGGCAGTATCACTC | GAAGGTCCACGGGAAAGACAC |
| TNF-α | GAAGGTCCACGGGAAAGACAC | CCACCACGCTCTTCTGTCTAC |
| IL-10 | GCTCTTACTGACTGGCATGAG | CGCAGCTCTAGGAGCATGTG |
| TGF-β | CCACCTGCAAGACCATCGAC | CTGGCGAGCCTTAGTTTGGAC |
| iNOS  GAPDH | ATCTTGGAGCGAGTTGTGGATTGTC  CAAGGCTGTGGGCAAGGTCATCC | TAGGTGAGGGCTTGGCTGAGTG  TTTCTCCAGGCGGCAGGTCAGAT |
