# Supplementary material for: Lactobacillus acidophilus and its metabolite ursodeoxycholic acid ameliorate ulcerative colitis by promoting Treg differentiation and inhibiting M1 macrophage polarization
Source: Front Microbiol. 2024 Jan 16;15:1302998. doi: 10.3389/fmicb.2024.1302998 (PMC10825044; doi:10.3389/fmicb.2024.1302998)
Supplement: Supplementary file 5 [file Table_2.DOCX]

**Table S2** **Identified different metabolites among groups in faces**

| Number | Differential metabolite | HMDB number | Molecular formula | Trend | Trend |
| --- | --- | --- | --- | --- | --- |
|  |  |  |  | DSS versus Control | LA versus DSS |
| 1 | 7-Ketodeoxycholic acid | HMDB0000391 | C_24_H_38_O_5_ | ↑ | ↓ |
| 2 | 3,7-Dihydroxy-12-oxocholanoic acid | HMDB0000400 | C_24_H_38_O_5_ | ↑ | ↓ |
| 3 | 3-Oxocholic acid | HMDB0000502 | C_24_H_38_O_5_ | ↑ | ↓ |
| 4 | 7Z,10Z-Hexadecadienoic acid | HMDB0000477 | C_16_H_28_O_2_ | ↓ | **↑** |
| 5 | Elaidic carnitine | HMDB0006464 | C_25_H_47_NO_4_ | ↑ | ↓ |
| 6 | MG(0:0/20:2(11Z,14Z)/0:0) | HMDB0011544 | C_23_H_42_O_4_ | ↑ | ↓ |
| 7 | Normetanephrine | HMDB0000819 | C_9_H_13_NO_3_ | ↑ | ↓ |
| 8 | Hydroxyvalerylcarnitine | HMDB0013132 | C_25_H_47_NO_5_ | ↑ | ↓ |
| 9 | MG(0:0/20:3(5Z,8Z,11Z)/0:0) | HMDB0011546 | C_23_H_40_O_4_ | ↑ | ↓ |
| 10 | Campesterol | HMDB0002869 | C_28_H_48_O | ↑ | ↓ |
| 11 | 5beta-Coprostanol | HMDB0000577 | C_27_H_48_O | ↑ | ↓ |
| 12 | PC(14:0/20:3(5Z,8Z,11Z)) | HMDB0007881 | C_42_H_78_NO_8_P | ↑ | ↓ |
| 13 | Tyrosyl-Valine | HMDB0029118 | C_14_H_20_N_2_O_4_ | ↓ | ↑ |
| 14 | PC(16:1(9Z)/22:5(4Z,7Z,10Z,13Z,16Z)) | HMDB0008022 | C_46_H_80_NO_8_P | ↑ | ↓ |
