# Supplementary material for: Lactobacillus acidophilus and its metabolite ursodeoxycholic acid ameliorate ulcerative colitis by promoting Treg differentiation and inhibiting M1 macrophage polarization
Source: Front Microbiol. 2024 Jan 16;15:1302998. doi: 10.3389/fmicb.2024.1302998 (PMC10825044; doi:10.3389/fmicb.2024.1302998)
Supplement: Supplementary file 6 [file Table_3.DOCX]

**Table S3 Criteria for the histological score of colon tissue.**

| Inflammatory cell infiltration | Score |
| --- | --- |
| Normal colonic tissue | 0 |
| Inflammatory or ulcerative lesions occur only in the mucosal layer | 1 |
| Inflammatory or ulcerative lesions appear only in the submucosa | 2 |
| Inflammation or ulcer deep lesions | 3 |
| Inflammation or ulceration of the lamina propria | 4 |
| Obvious perforated ulcers | 5 |
